# Association of Grip Strength with Quality of Life in the Chinese Oldest Old

**DOI:** 10.3390/ijerph182312394

**Published:** 2021-11-25

**Authors:** Boqin Xie, Chenjuan Ma

**Affiliations:** 1 School of Nursing, Fudan University, 305 Rd. Fenglin, Shanghai 200032, China; 2 Rory Meyers College of Nursing, New York University, New York, NY 10010, USA; cm4215@nyu.edu

**Keywords:** quality of life, grip strength, elderly, WHOQOL-OLD, oldest old, community dwellers

## Abstract

Emerging studies have suggested an association between grip strength and health-related quality of life (QOL). However, evidence for which specific domains of QOL are associated with grip strength remains limited and inconsistent. Particularly, such evidence is scarce in the oldest old, who constitute one of the most vulnerable populations. This cross-sectional study aimed to examine the association between grip strength and overall QOL as well as specific domains in the oldest old. It included 400 community-dwelling older adults aged 80 years or older from Shanghai, China. QOL was assessed using the WHO Quality of Life of Older Adults instrument, and grip strength was measured using a digital spring-type dynamometer. On average, the overall QOL score was 54.68 (SD = 12.05). Estimates of risk-adjusted linear regressions indicated that higher grip strength was associated with better overall QOL (*β* = 4.40, *p* < 0.001) as well as the domains of autonomy (*β* = 6.74, *p* < 0.001); fulfillment with past, present, and future activities and achievements (*β* = 3.52, *p* = 0.004); and satisfaction with social participation (*β* = 6.72, *p* < 0.001). Our findings highlight the importance of maintaining or improving grip strength in delaying or reducing the decline in QOL among the community-dwelling oldest old. Also noteworthy is that the associations between grip strength and specific domains of QOL in the oldest old vary.

## 1. Introduction

The oldest old, aged 80 years or above, have become the fastest-growing population segment around the world due to improvements in economic and social conditions, and ongoing medical advances [1,2]. Worldwide, the number of people in the oldest old population has been projected to triple by 2050, growing from 126.5 million in 2015 to 446.6 million in 2050 [3]. In China, this population reached 35.8 million in 2020 and is expected to continue to grow rapidly [4].

As life expectancy increases, improving quality of life (QOL) and maintaining the wellbeing of older adults, particularly the oldest old for as long as possible, has become increasingly important [5]. These are also the optimal goals of individuals, communities, and nations [6]. QOL, a multidimensional construct of a person’s general status of wellbeing, is defined by the World Health Organization (WHO) as “an individual’s perceptions of their position in life, in the context of the culture and value system in which they live, and in relation to their goals, expectations, standards and concerns” [7]. QOL has been recognized as an indicator of unmet needs in older adults and disease-based outcomes and is used to estimate the efficacy of health services and interventions [8]. People who are among the oldest old are particularly vulnerable to having poor QOL due to the increased risks of chronic diseases, physical disabilities, cognitive impairments, and mental disorders with advanced age [9]. Hence, a better understanding of the factors, particularly modifiable factors, associated with QOL of the oldest old is critical for identifying potential interventions for improving QOL. 

Emerging studies have demonstrated that grip strength is a modifiable factor associated with quality of life in older adults. Grip strength is a simple, effective, and noninvasive test of upper limb strength measured using a hand dynamometer. It has been recognized as an important marker of physical frailty, sarcopenia, and malnutrition in recent studies [10,11,12]. In a study of Chinese older adults with a mean age of 70 years, Yang et al. [13] found that lower grip strength was associated with reduced overall health-related quality of life (HRQoL), measured using the Euro Quality of Life Visual Analogue Scale. Laudisio et al. [14] reported that grip strength was associated with both the physical functioning domain and the mental health domain of HRQoL measured using the Medical Outcomes Study Form 12 (SF-12) among community-dwelling older adults. However, Sayer et al. [15] did not find an association between grip strength and the mental health domain of HRQoL assessed using the SF-36. A similar result was reported by Haider et al. [16], who observed the associations between grip strength and overall QOL using the World Health Organization Quality of Life-BREF assessment (WHOOQL-BREF), and the autonomy domain using the World Health Organization Quality of Life Instrument-Older Adults Module (WHOQOL-OLD) but not with other domains of WHOOQL-BREF and WHOQOL-OLD, among older adults. In addition to the inconsistency in evidence reported, notably, previous studies have mainly focused on the relationship between grip strength and the physical and mental health aspects of QOL, whereas few studies have investigated the associations between grip strength and other aspects of QOL (e.g., sensory abilities, autonomy, or perception of death and dying), despite their high relevance to the QOL and wellbeing of people who are older [17]. Additionally, a scarce number of studies focus on the oldest old group, particularly those living in China. Clearly, the relationship between grip strength and QOL, especially specific domains of QOL, needs to be better understood. 

Therefore, using the instrument WHOQOL-OLD, which was specifically developed for assessing QOL in older adults, the purpose of this study was to examine the association between grip strength and QOL and to determine which specific domains of QOL are affected by grip strength among Chinese community-dwelling oldest old. We hypothesize that (1) higher grip strength is associated with better QOL and (2) the magnitude of the association between grip strength and QOL varies across domains of QOL. 

## 2. Materials and Methods

### 2.1. Study Design and Participants

This study used a cross-sectional design and was conducted from October 2020 to March 2021 in Shanghai, China. The convenience cluster sampling method was used for recruiting and selecting participants. First, four neighborhoods were selected from the Da-Chang Subdistrict of Baoshan District using the convenience sampling method. Second, we reached out to all older adults aged 80 years or older who registered at primary healthcare centers (Family Doctor Service Plan centers) in the aforementioned neighborhoods for recruitment. The inclusion criteria of this study were being an older adult living in the community, an age of 80 years or older, no clinical diagnosis of dementia, no severe or unstable stage of somatic or psychiatric diseases, and willingness to participate in the study. Older adults who had severe visual and/or hearing impairments or had injuries, surgery, or acute diseases of an upper extremity in the past 6 months and those who were unable to perform the grip test were excluded. Initially, 445 older adults were contacted. Five of them refused to participate, and forty were excluded because of severe somatic diseases (*n* = 11), visual/hearing impairments (*n* = 15), dementia diagnosis (*n* = 8), and incomplete data on QOL (*n* = 6). Finally, a total of 400 eligible older adults were included in this study. 

A face-to-face interview modality was used to collect the data. Data collection was conducted by trained research assistants at the participants’ homes. Written informed consent was obtained from all participants during the home visit before data collection. This study was approved by the Ethics Committee of the authors’ institute (reference number: IRB#TYSQ 2019-5-02). 

### 2.2. Measures

#### 2.2.1. Quality of Life

Quality of life was assessed using the WHO Quality of Life of Older Adults (WHOQOL-OLD) scale, which was designed to assess the generic QOL of adults aged 60 years or older [18]. This scale has been translated into Chinese and has demonstrated good psychometric properties in Chinese older adults [19]. The scale consists of 24 items in six domains, including (1) sensory functioning (SAB, four items), referring to the impact of impairments in sensory functioning on daily life; (2) autonomy (AUT, four items), measuring the extent of being able or being free to live autonomously and to make own decisions; (3) past present and future activities (PPF, four items), assessing the degree of satisfaction for achievements in life as well as a general future outlook; (4) social participation (SOP, four items), referring to engagement in activities in the community; (5) death and dying (DAD, four items), assessing the degree of concerns, worries, and fears about death and dying; and (6) intimacy (INT, four items), examining the level of satisfaction with personal and intimate relationships. Responses were rated on a 5-point Likert scale from 1, representing absolute disagreement, to 5, representing absolute agreement. The scores for the WHOQOL-OLD total and for each of the six domains were summed and then transformed based on standard algorithms into scores ranging from 0 to 100 [20]. Higher scores indicated better quality of life. The reliability of the scale was Cronbach’ alpha = 0.938, and Cronbach’ alpha values of each domain ranged from 0.722 to 0.949 in this study, which indicated good reliability. 

#### 2.2.2. Grip Strength

Grip strength was measured in kilogram using a digital spring-type dynamometer (Camry EH10; Sensun Weighing Apparatus Group Ltd., Guangdong, China) following the procedure recommended by the Asian Working Group for Sarcopenia (AWGS) in 2019 [12]. Before data collection, the dynamometers were calibrated to minimize measurement errors. During data collection, participants were asked to squeeze the dynamometer with their dominant hand as hard as possible for at least 5 s in a standing position with full elbow extension. If a participant could not stand without assistance, they were allowed to sit down to perform this task. Each participant performed the task twice, with a 30 s interval. Measures of grip strength were reported as the maximum value of two measurements with the dominant hand. Due to the significant gender difference in grip strength [21], the participants in each gender group were divided into three groups according to gender-stratified tertiles of grip strength in this study: low grip strength (11.2–21.2 kg for males; 4.8–13.7 kg for females), middle grip strength (21.3–27.7 kg for males; 14–18.5 kg for females), and high grip strength (27.9–40.2 kg for males; 18.6–28.8 kg for females). 

#### 2.2.3. Covariates 

In this study, a set of variables that are associated with the quality of life of older adults in the existing literature was taken into account and included as covariates in this study, including sociodemographics and factors reflecting clinical and health conditions of the participants. The sociodemographic data included age, gender, education (9 years or less of schooling, or more), marriage status (married/unmarried, widowed, or divorced), and living arrangement (living alone/living with others). To measure the number of chronic conditions, participants were asked if they were diagnosed with any of the following chronic conditions: hypertension, diabetes, stroke, cardiac diseases, chronic lung diseases, digestive diseases, liver disease, kidney diseases, arthritis/rheumatism, Parkinson’s disease, and cancer. Participants were also asked how many types of medications they took regularly; taking five or more types of medications was considered polypharmacy. To measure limitations in basic or instrumental activities of daily living (ADL/IADL), participants were asked if they were able to independently perform any of the following daily activities: dressing, eating, toileting, bathing, grooming, transfers, using the telephone, grocery shopping, preparing meals, housekeeping, laundry, driving or using public transportation, administering own medication, and handling money and goods [22]. Depression was measured using the five-item version of the Geriatric Depression Scale (GDS-5), and participants with a GDS-5 score of 2 or above were considered having depressive disorder [23]. The six-item UCLA Loneliness Scale was used to assess the extent of loneliness, and higher scores indicated greater loneliness [24]. Cognitive function was assessed using the Chinese version of the Montreal Cognitive Assessment Basic (MOCA-B), and the education-stratified cut-off points were used to identify whether a participant had cognitive impairment [25]. 

### 2.3. Data Analysis

Descriptive statistics were used to summarize the distribution of each study variable, including frequency and percentage for categorical variables, mean and standard deviation for continuous variables, and median and interquartile range for nonnormally distributed variables. Bivariate analyses were then conducted to compare the demographics, health conditions, psychosocial factors, and grip strength between older adults with low overall QOL and those with high overall QOL. Participants had low overall QOL if their overall WHOQOL-OLD scores were below the median value. More specifically, in the bivariate analysis, Chi-square tests were used for categorical variables and *t* tests or Mann–Whitney U-tests were used for continuous variables, depending on the distribution. A radar plot was used to visually illustrate the differences in the six domains of the WHOQOL-OLD scores across gender-stratified tertiles of grip strength. 

To test the association between grip strength and QOL, linear regression models were used with WHOQOL-OLD scores overall and for each domain. Grip strength was classified into three groups based on gender-stratified tertiles. The models were adjusted by sociodemographics, health conditions, and psychosocial factors as covariates. Specifically, Model 1 was adjusted for sociodemographics and health conditions only, including age, gender, education, marital status, living arrangement, number of chronic conditions, polypharmacy, and functional limitations. Model 2 further included depressed symptoms, loneliness, and cognitive function on top of those in Model 1. In the regression models, partial ω^2^ was used to present relevant estimates of effect size, which indicated the proportion of outcome variables (QOL overall and for each domain) explained by the explanatory variable (grip strength) after excluding the effect of other factors in the same model. The magnitude of ω^2^ is generally classified as follows: up to 0.06 is considered a small effect, from 0.06 to 0.14 is considered a medium effect, and 0.14 or greater is a large effect [26]. All tests were two-sided, with a *p* value of <0.05 being considered statistically significant. All data management and analyses were performed using Stata 14 (Stata Corp, College Station, TX, USA).

## 3. Results

### 3.1. Sample Characteristics

A total of 400 older adults aged 80 years or above (mean age 85.18 ± 4.20; 47.04% females) were included in this study. Over half of the participants had 9 years or less of schooling (52.25%), had three or more chronic conditions (55.25%), or were impaired in cognitive function (62%). Nearly 40% of older adults took five or more types of medications regularly (39.25%), had limitations in ADL/IADL (45.75%), and had depressive disorder (40.50%). Details of the sample characteristics are shown in Table 1. 

Of the 400 older adults, 49.2% reported high overall QOL, with a WHOQOL-OLD score above the median score (54 points). A comparison of the participant characteristics between the high overall QOL and low QOL groups is presented in Table 1. Compared with those with high overall QOL (≥54 points), older adults with low overall QOL (<54 points) were more likely to be older (*p* < 0.001), to be unmarried (*p* = 0.001), to have more chronic conditions (*p* < 0.001), to take five or more type of medications (*p* < 0.001), to experience ADL/IADL limitations (*p* < 0.001), to be depressed (*p* < 0.001), to be lonely (*p* < 0.001), and to have cognitive impairment (*p* < 0.001). The average grip strength of our sample was 19.82 ± 6.94 Kg. Older adults with low overall QOL had weaker grip strength than those with high overall QOL (17.07 ± 6.47 vs. 22.65 ± 6.25, *p* < 0.001).

### 3.2. Association between Grip Strength and Quality of Life 

**Figure 1:**
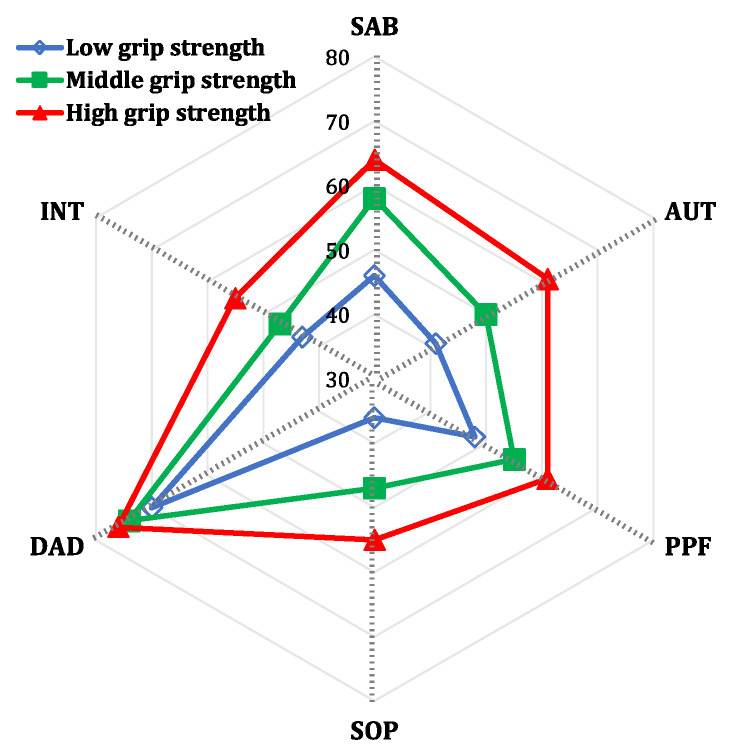
A radar plot on the WHOQOL-OLD scores for all six domains by the tertiles for grip strength. Note: SAB = sensory ability; AUT = autonomy; PPF = past, present, and future activities; SOP = social participation; DAD = perception of death and dying; INT = intimacy.

In our sample, the mean overall WHOQOL-OLD score for the participants was 54.68 ± 12.05. The death and dying domain had the highest mean score (73.21 ± 13.98), and the social participation domain had the lowest mean score (45.87 ± 16.40). A radar plot shows the WHOQOL-OLD scores for all six domains according to grip strength groups (Figure 1). Older adults in the high grip strength group had better QOL for all domains, except for the death and dying domain, which had similar scores across grip strength group. 

Table 2 summarizes the results of linear regression models, estimating the association between grip strength and QOL, overall and by each domain, and with and without adjustment for the covariates. A significant association between grip strength and overall QOL was identified. Compared with older adults with low grip strength, those with middle grip strength (*β* = 2.04, *p* = 0.027) and high grip strength (*β* = 4.40, *p* < 0.001) had greater overall QOL after adjustment of potential confounders. Additionally, a significant, positive linear trend in the association between grip strength and overall QOL was observed even after risk adjustment (*p* for trend < 0.001). The proportion of variance explained by the differences in grip strength on the overall QOL was 5.3% after the full adjustments.

The results of the linear regression models estimating the relationship between grip strength and each domain of the QOL (Table 2) indicate that higher grip strength was significantly associated with greater QOL in the domains of AUT (*β* = 6.74, *p* < 0.001), PPF (*β* = 3.52 *p* = 0.004), and SOP (*β* = 6.72 *p* < 0.001) after the adjustment for demographics, health conditions, and psychosocial covariates. Additionally, significant linear trends in the association between increasing grip strength and better AUT, PPF, and SOP were observed (*p* for trend <0.05). The partial ω^2^ attributable to the variation in grip strength was over 0.06 for the SOP domain, which indicates a medium effect size. 

As shown in Table 2, significant positive associations of grip strength with the QOL domains of SAB, DAD, and INT were observed in the crude models and in the models adjusted for the demographics and health conditions. However, these associations were not statistically significant when further adjusted for the psychosocial covariates, including depression, loneliness, and cognitive impairment (Model 2). 

## 4. Discussion

The findings in this study indicated that stronger grip strength was associated with an increase in perceived quality of life in Chinese community-dwelling oldest old. Furthermore, this study specified the domains of QOL, including autonomy; past, present, and future activities; and social participation that are significantly associated with grip strength. 

Despite the use of various operationalizations of QOL measurement in research to date, the results of our study are consistent with those from previous studies that also reported a significant association between grip strength and the overall QOL in older adults [13,15,16]. Older adults with weak hand-grip strength may have difficulties in completing multiple activities in daily life such as grocery shopping, preparing meals, or housekeeping, which are critical parts of daily living activities. Previous studies have demonstrated that limitations in activities in daily life were associated with poor QOL in older adults [27,28], which may be one possible reason underlying the association between weak grip strength and poor QOL. Another potential mechanism of the relationship between low grip strength and QOL may be the impaired mental health caused by low grip strength [29,30], as previous studies have shown that mental health is one of most important factors influencing quality of life in older adults [27,28,31]. Our study indicated that grip strength is an important factor independently associated with overall QOL in the oldest old, which implies that primary care providers can screen grip strength to identify people among the oldest old who are vulnerable to, and at risk for, poor overall QOL. Our study also suggests that future studies are needed to develop an intervention that enhances muscle strength in order to improve QOL of the oldest old who are unable to perform high-intensity oxygen exercise. 

Our study found that the magnitude of association between grip strength and QOL differed across domains of QOL. In our study, small-to-medium effect sizes of grip strength were found on the QOL domains related to autonomy; fulfillment with past, present, and future activities and achievements; and satisfaction with social participation. Indeed, this association well illustrates the negative consequences of physical limitations on older adults’ QOL. Autonomy is the capacity of an individual to independently control, cope with, and make decisions about his or her daily life [32]. Retaining autonomy in daily life largely relies on one’s physical fitness and wellness [33]. Likewise, physical fitness is crucial for older adults to engage in social participation and to maintain expectations for future achievements. While grip strength is a direct measure of upper extremity strength, it has often been recognized as a proxy of whole-body muscle strength, which is an important component of physical fitness [10,11]. These may be the underlying mechanisms of the association between grip strength and the aforementioned three domains of QOL. 

In addition, regarding the QOL domains of perceptions of sensory ability, death and dying, and intimacy, our results using hierarchical regression models showed that their associations with grip strength were not statistically significant after the adjusting for depression, loneliness, and cognitive impairment. Our findings suggest that grip strength, often used a proxy of muscle strength, may not be a critical factor influencing sensory ability, death and dying, and intimacy in QOL. These results, on the one hand, suggest that psychosocial factors may mediate the associations among grip strength and perceptions of sensory ability, death and dying, and intimacy. Future studies are warranted to identify the potential mediating effect of these factors in order to explain the domain-specific association between QOL and grip strength. On the other hand, our findings imply that mental health and cognitive ability play important roles in those three domains of QOL (Supplemental Data, Table S1). Therefore, in addition to assessing the physical performance of grip strength and common chronic conditions, healthcare providers should pay attention to the mental health and cognitive status of older adults in order to better monitor their QOL and its changes.

Our findings in this study also highlight the urgency to improve QOL for the community-dwelling oldest old. The transformed scale score of WHOQOL-OLD among older adults who participated in our study was 54.64, which is much lower than that reported by other researchers using the same scale of QOL. Using the transformed scale score of WHOQOL-OLD among older adults aged 60 years or above, Zhang et al. [34] reported that the overall QOL score of urban older adults living in Xi’an, China, was 74.28. Internationally, Moreno-Tamayo et al. [35] reported a score of 68.5 among Mexican older adults, and Gobbens et al. [36] reported a score of 91.7 among Dutch older adults. One possible reason for these differences may be the older age of the participants in our study, as advanced age is a risk factor of poor QOL, established in previous studies [37,38]. In addition, our findings that Chinese oldest old had especially poor QOL in the domains of satisfaction with social participation and perception of intimacy suggest that more opportunities to participate in social activities and tailored interventions to boost intimate relationships should be provided to the oldest old to promote their quality of life. 

Several limitations in this study should be noted. First, due to a cross-sectional design, we were unable to identify the causality between grip strength and QOL in the oldest old. Longitudinal studies are needed in the future to determine the direction of the links between weak grip strength and poor QOL. Second, caution should be taken when generalizing our conclusions to other oldest old population in China or other nations, given that this study was conducted in one region of China, Shanghai. Additionally, a sample size of 400 in this study was relatively small given the total population in Shanghai and China. However, our post hoc power analysis using G*Power 3.1 software based on the sample size obtained and parameter estimates of grip strength derived from the current study and two-tailed test with an α = 0.05 level achieved a power of 0.996, which indicates that the sample size in this study was adequate. Third, although we controlled for some demographics, health conditions, and psychosocial covariates that may influence the QOL of older adults, some unmeasured confounders may have influenced the results. 

## 5. Conclusions

The findings of this study contribute to the growing body of evidence on the relationship between weak grip strength and the risk of declining QOL among the community-dwelling oldest old. In addition, our results showed that different domains of QOL were affected by grip strength differently. These findings highlighted the importance of improving or maintaining the grip strength via various approaches, such as resistance training, in maintaining QOL among older adults, including the oldest old. These findings also suggest that health providers may consider using grip strength measurement as a simple and reliable screening test for an initial assessment of quality of life in the community-dwelling oldest old. Moreover, to improve the QOL from a holistic approach, both enhancing physical function and maintaining mental health and cognitive function are important for the oldest old.

## Figures and Tables

**Table 1:** Sample characteristics based on a median split of the overall WHOQOL-OLD score (*n* = 400).

		Total (*n* = 400)	Low QOL (*n* = 203)	High QOL (*n* = 197)	*p* Value
		N (%)/Mean (SD)/Median (IQR)	N (%)/Mean (SD)/Median (IQR)	N (%)/Mean (SD)/Median (IQR)	
Age (years)		85.18 (4.20)	86.66 (4.31)	83.70 (3.53)	<0.001 **
Age group	80–85 years	228 (57.00)	86 (42.36)	142 (72.08)	<0.001 **
	≥86 years	172 (43.00)	117 (57.64)	55 (27.92)	
Gender	Female	186 (46.5)	85 (41.87)	101 (51.27)	0.060
	Male	214 (53.50)	118 (58.13)	96 (48.73)	
Educated years	≤9 years	209 (52.25)	114 (56.16)	95 (48.22)	0.112
	>9 years	191 (47.75)	89 (43.84)	102 (51.78)	
Marital status	unmarried	163 (40.75)	99 (48.77)	64 (32.49)	0.001 *
	Married	237 (59.25)	104 (51.23)	133 (67.51)	
Living arrangement	Alone	60 (15.00)	30 (14.78)	30 (15.23)	0.900
	With others	340 (85.00)	173 (85.22)	167 (84.77)	
Number of chronic conditions	none or one	83 (20.75)	19 (9.36)	64 (32.49)	<0.001 **
	two	96 (24.00)	35 (17.24)	61 (30.96)	
	≥three	221 (55.25)	149 (73.40)	72 (36.55)	
Polypharmacy	No	243 (60.75)	83 (40.89)	160 (81.22)	<0.001 **
	Yes	157 (39.25)	120 (59.11)	37 (18.78)	
ADL/IADL limitations	Normal	217 (54.25)	46 (22.66)	171 (86.80)	<0.001 **
	Limited	183 (45.75)	157 (77.34)	26 (13.20)	
Depression	Normal	238 (59.50)	71 (34.98)	167 (84.77)	<0.001 **
	depressed	162 (40.50)	132 (65.02)	30 (15.23)	
Loneliness (score) ^a^		7 (4)	9 (7)	6 (1)	<0.001 **
Cognitive function	Normal	152 (38.00)	36 (17.73)	116 (58.88)	<0.001 **
	Impaired	248 (62.00)	167 (82.27)	81 (41.12)	
Handgrip strength		19.82 (6.94)	17.07 (6.47)	22.65 (6.25	<0.001 **

Note: IQR = interquartile range; * *p* < 0.05, ** *p* < 0.001; ^a^ variable described as median and IQR.

**Table 2:** Multiple linear regression analyses for the associations between handgrip strength and WHOQOL-OLD, overall and in each domain (*n* = 400).

Main Independent Variable	Crude Model		Adjusted Model1 ^a^		Adjusted Model 2 ^b^	
	β (95%CI)	*p*	β (95%CI)	*p*	β (95%CI)	*p*
***Overall QOL***						
Grip strength						
Low	Ref.		Ref.		Ref.	
Medium	7.86 (5.36, 10.37)	<0.001	3.26 (1.18, 5.34)	0.002	2.04 (0.23, 3.85)	0.027
High	14.82 (12.31, 17.33)	<0.001	6.96 (4.69, 9.22)	<0.001	4.40 (2.40, 6.40)	<0.001
*p* for trend	<0.001		<0.001		<0.001	
partial ω^2^	0.336		0.104		0.053	
***Sensory ability***						
Grip strength						
Low	Ref.		Ref.		Ref.	
Medium	12.42 (8.11, 16.72)	<0.001	5.59 (1.41, 9.78)	0.009	4.00 (−0.01, 8.02)	0.051
High	18.35 (14.03, 22.66)	<0.001	7.44 (2.89, 11.99)	0.001	4.29 (−0.14, 8.73)	0.058
*p* for trend	<0.001		0.002		0.063	
partial ω^2^	0.219		0.038		0.004	
***Autonomy***						
Grip strength						
Low	Ref.		Ref.		Ref.	
Medium	9.50 (5.97, 13.03)	<0.001	2.84 (−0.09, 5.77)	0.058	1.70 (1.87, 7.70)	0.231
High	20.53 (16.99, 24.07)	<0.001	9.17 (5.98, 12.36)	<0.001	6.74 (3.66,9.81)	<0.001
*p* for trend	<0.001		<0.001		<0.001	
partial ω^2^	0.307		0.079		0.044	
***Past, present and future activities***						
Grip strength						
Low	Ref.		Ref.		Ref.	
Medium	6.66 (4.09, 9.22)	<0.001	2.81 (0.42, 5.19)	0.021	1.62 (−0.52, 3.76)	0.136
High	12.94 (10.37, 15.52)	<0.001	6.13 (3.53, 8.72)	<0.001	3.52 (1.15, 5.88)	0.004
*p* for trend	<0.001		<0.001		0.004	
partial ω^2^	0.254		0.061		0.022	
***Social participation***						
Grip strength						
Low	Ref.		Ref.		Ref.	
Medium	11.06 (7.63, 14.49)	<0.001	5.08 (2.18, 7.98)	0.001	3.60 (0.91, 6.29)	0.009
High	19.72 (16.28, 23.16)	<0.001	9.43 (6.28, 12.59)	<0.001	6.72 (3.75,9.69)	<0.001
*p* for trend	<0.001		<0.001		<0.001	
partial ω^2^	0.329		0.106		0.061	
***Perception of death and dying***						
Grip strength						
Low	Ref.		Ref.		Ref.	
Medium	3.61 (0.29, 6.92)	0.033	2.98 (−0.43, 6.40)	0.086	2.41 (−0.99, 5.82)	0.164
High	5.41 (2.09, 8.73)	0.001	4.14 (0.46, 7.85)	0.029	2.85 (−0.91, 6.62)	0.971
*p* for trend	0.001		0.530		0.970	
partial ω^2^	0.031		0.031		0.142	
***Intimacy***						
Grip strength						
Low	Ref.		Ref.		Ref.	
Medium	3.94 (0.42, 7.47)	0.0281	0.24 (−3.22, 3.71)	0.891	−1.09 (−4.28, 2.10)	0.503
High	11.98 (8.45, 15.51)	<0.001	5.44 (1.68, 9.21)	0.005	2.29 (−1.23, 5.82)	0.202
*p* for trend	<0.001		0.004		0.183	
partial ω^2^	0.107		0.015		0.0002	

Note: *β* = unstandardized regression coefficient; CI = confidence interval; ω^2^ = omega squared; Ref. = reference group. ^a^ Model 1 adjusted for age, gender, education, marital status, living arrangement, number of chronic conditions, polypharmacy, and functional limitations. ^b^ Model 2 adjusted for demographics, health conditions, and psychosocial factors, including age, gender, education, marital status, living arrangement, number of chronic conditions, polypharmacy, functional limitations, depressed symptoms, loneliness, and cognitive function.

## Supplementary Materials

The following are available online at https://www.mdpi.com/article/10.3390/ijerph182312394/s1, Table S1: Results of the fully adjusted models on the associations of grip strength overall and specific domains of QOL.
