# Supplementary material for: Association of Grip Strength with Quality of Life in the Chinese Oldest Old
Source: Int J Environ Res Public Health. 2021 Nov 25;18(23):12394. doi: 10.3390/ijerph182312394 (PMC8656506; doi:10.3390/ijerph182312394)
Supplement: Supplementary file 1 [file ijerph-18-12394-s001.zip › ijerph-1452349-SI.pdf]

**Table S1.** Results of the fully adjusted models on the associations of grip strength with overall and specific domain of QOL.							
	**Overall QOL**	**SAB**	**AUT**	**PPF**	**SOP**	**DAD**	**INT**
	β **(95%CI)**	***β*** **(95%CI)**	***β*** **(95%CI)**	***β*** **(95%CI)**	***β*** **(95%CI)**	***β*** **(95%CI)**	***β*** **(95%CI)**
Middle grip strength (vs. low grip strength)	**2.04** (0.23, 3.85)	4.01 (-0.01. 8.02)	1.70 (-1.08, 4.48)	1.62 (-0.52, 3.76)	**3.60** (0.91, 6.29)	2.41 (-0.99, 5.82)	-1..09 (-4.28, 2.10)
High grip strength (vs. low grip strength)	**4.40** (2.40, 6.40)	4.29 (-0.14, 8.73)	**6.73** (3.66, 9.81)	**3.52** (1.15, 5.88)	**6.72** (3.75, 9.69)	2.86 (-0.91, 6.62)	2.09 (-1.23, 5.82)
Age	-0.09 (-0.29, 0.12)	**-0.51** (-0.96, -0.06)	-0.14 (-0.46. 0.17)	-0.15 (-0.39, 0.09)	-0.12 (-0.43, 0.18)	**0.52** (0.14, 0.91)	-0.11 (-0.46, 0.25)
Female (vs. male)	-0.54 (-2.19, 1.11)	1.53 (-2.13, 5.19)	-0.11 (-2.65, 2.42)	-0.68 (-2.64, 1.27)	-2.11 (-4.56, 0.34)	-1.61 (-4.72, 1.50)	-0.27 (-3.18, 2.64)
Education: Middle school (vs. elementary school or less)	1.16 (-0.71, 3.03)	1.80 (-2.36, 5.96)	0.88 (-2.00, 2.42)	**3.80** (1.59, 6.01)	1.29 (-1.49, 4.08)	-0.95 (-4.47, 2.58)	0.14 (-3.16, 3.44)
Education: Associate degree or college or above (vs. elementary school or less0	1.56 (-0.61, 3.73)	2.41 (-2.39, 7.22)	2.78 (-1.05, 5.61)	**3.58** (1.02, 6.14)	2.56 (-0.66, 5.77)	-2.42 (-6.51, 1.65)	0.98 (-2.84, 4.80)
Married (vs. unmarried)	-0.06 (-1.84, 1.73)	2.64 (-1.32, 6.60)	0.03 (-2.71, 2.78)	-1.74 (-3.85, 0.37)	-2.30 (-4.95, 0.35)	-0.69 (-4.05, 2.67)	1.72 (-1.43, 4.87)
Living alone (vs. living with others)	-0.04 (-2.28, 2.20)	0.31 (-4.65, 5.27)	1.43 (-2.01, 4.87)	0.43 (-2.22, 3.07)	-0.75 (-4.07, 2.57)	3.40 (-0.81, 7.61)	**-5.04** (-8.99, -1.10)
Comorbidity: Two (vs. one or less)	-1.96 (-4.06, 0.14)	0.25 (-4.40, 4.90)	-3.61 (-6.83, -0.38)	-1.16 (-3.64, 1.32)	-1.52 (-4.64, 1.59)	-2.74 (-6.69, 1.21)	-2.97 (-6.67, 0.72)
Comorbidity: Three or	-1.81	-0.90	**-3.57**	0.92	-1.42	**-4.16**	-1.71

more (vs. one or less)	(-3.91, 0.29)	(-5.56, 3.77)	(-6.80, -0.34)	(-1.56, 3.41)	(-4.54, 1.70)	(-8.12, -0.21)	(-5.41,1.99)
Polypharmacy (vs. no)	-4.14	-3.16	**-4.36**	**-3.47**	**-5.34**	**-3.72**	**-4.80**
	(-5.82, -2.47)	(-6.87, 0.55)	(-6.93, -1.78)	(-5.44, -1.49)	(-7.83, -2.86)	(-6.87, -0.57)	(-7.75, -1.85)
ADL/IADL disabilities (vs.	**-7.22**	**-7.58**	**-12.91**	**-5.73**	**-11.03**	-2.79	**-3.29**
Independency)	(-9.06, -5.38)	(-11.65, -3.50)	(-15.73, -10.08)	(-7.90, -3.56)	(-13.75, -8.30)	(-6.25, 0.67)	(-6.52, -0.05)
Depressed (vs. normal)	**-4.35**	**-6.39**	**-3.78**	**-3.78**	**-6.99**	-1.77	**-3.43**
	(-6.05. -2.67)	(-10.14, -2.64)	(-6.38, -1.19)	(-5.77, -1.78)	(-9.50, -4.48)	(-4.94, 1.41)	(-6.41, -0.46)
Loneliness	**-0.71**	-0.49	**-0.45**	-0.89	**-0.49**	**-0.50**	**-1.42**
	(-0.94. -0.47)	(-0.99,0.02)	(-0.80, -0.10)	(1.16, -0.62)	(-0.83, -0.15)	(-0.94, -0.07)	(-1.83, -1.02)
Cognitive impairment	**-4.79**	**-7.76**	**-6.51**	**-4.28**	**-4.87**	-1.67	**-3.63**
(vs. normal)	(-6.34, -3.23)	(-11.20, -4.30)	(-8.90, -4.11)	(-6.12, -2.44)	(-7.19, -2.56)	(-4.61, 1.26)	(-6.38, -0.89)

Note: Bold font indicating $p<0.05$;SAB=sensory functioning; AUT=autonomy; PPF=past, present and future activities; SOP=social participation; DAD=death and dying; INT=intimacy.
